# Clinical Features and Rules of Chinese Herbal Medicine in Diabetic Peripheral Neuropathy Patients

**DOI:** 10.1155/2020/5795264

**Published:** 2020-07-17

**Authors:** Jindong Zhao, Yan Li, Ling Xin, Min Sun, Chanjuan Yu, Guobin Shi, Taotao Bao, Jian Liu, Yingqun Ni, RuiMin Lu, Yuanyuan Wu, Zhaohui Fang

**Affiliations:** ^1^Department of Endocrine Disease, The First Affiliated Hospital of Anhui University of Chinese Medicine, Hefei 230031, China; ^2^Department of Infectious Disease, The First Affiliated Hospital of Anhui University of Chinese Medicine, Hefei 230031, China; ^3^Department of Information, The First Affiliated Hospital of Anhui University of Chinese Medicine, Hefei 230031, China; ^4^School of Life Sciences, Anhui University, Hefei 230039, China

## Abstract

**Objective:**

To analyse the clinical features of diabetic peripheral neuropathy (DPN) and employ data mining technology to explore the rules of Chinese herbal medicine (CHM) therapy.

**Methods:**

The clinical data of 216 patients with DPN and qi-yin deficiency syndrome were obtained, and the clinical features of the patients were assessed by cluster analysis. Relevant information was entered into the clinical diagnosis and treatment collection system, and data mining techniques were used to analyse the drug frequency, core CHM, CHM pair, and so on.

**Results:**

In this study, glycated haemoglobin (HbA1c) and homocysteine (HCY) were closely related to the pathogenesis of DPN. Overall, 162 patients had typical DPN syndrome characteristics, and we analysed 216 prescriptions, including 182 CHM. The frequencies of prescription of *Astragalus membranaceus*, *Ligusticum wallichii*, *Poria cocos*, and Radix Rehmanniae were greater than 45%. A Bayesian network analysis diagram showed that the 9 most common core CHM included *Astragalus membranaceus*, *Ligusticum wallichii*, *Poria cocos*, atractylodes rhizome, and *Salvia miltiorrhiza* Bge. According to the association rules of CHM, Radix Ophiopogon is used for Codonopsis pilosula; *Astragalus membranaceus* and atractylodes rhizome for *Rehmannia* are also frequently used. *Astragalus membranaceus* and Cinnamomi Ramulus or *Ligusticum wallichii* and Moutan bark were highly related to a decreased Michigan Diabetic Neuropathy Score.

**Conclusion:**

HbA1c and HCY are related risk factors for DPN. Numbness is a typical syndrome characteristic. *Astragalus membranaceus* is a monarch CHM and is used most frequently.

## 1. Introduction

The incidence of diabetic peripheral neuropathy (DPN) is approximately 10–96% and is one of the more common chronic complications of diabetes [1]. The main causes of DPN are metabolic disorders and vascular damage [2]. DPN presents as a wide spectrum of symptoms, such as sensory loss, pain, and loss of muscle mass [3]. Although nerve conduction velocity is common in DPN patients, it is not easily or reliably diagnosed in DPN [4]. However, 10 g of nylon yarn, a 128 Hz tuning fork, the Leeds Assessment of Neuropathic Symptoms and Signs, the Toronto Clinical Scoring System, and other tools have been widely used in the diagnosis of DPN [5–8]. Currently, the Michigan Diabetic Neuropathy Score (MDNS) is also a commonly used tool for diagnosing DPN [9].

Some research has proven the efficacy of Chinese herbal medicine (CHM) in the treatment of DPN [10–12]. Thus, we believe that Traditional Chinese Medicine (TCM) has potential in the prevention and treatment of DPN. The pathogenesis of DPN is qi-yin deficiency in TCM, and this pattern gradually develops into a yin and yang deficiency. Blood stasis is a sign in the blood vessels and results in pain and itching [13]. We have recorded the general information of DPN patients and the prescription of CHM treatment based on real-world applications. This study provides a summary of patients' clinical characteristics and experience with CHM.

## 2. Subjects and Methods

### 2.1. Patient Recruitment

The patients selected for this study included 216 patients with DPN with qi-yin deficiency syndrome who were treated with CHM, which included 216 prescriptions, from January 2016 to December 2019 at the First Affiliated Hospital of Anhui University of Chinese Medicine. Patients for whom treatment was effective were included. The main basis for evaluating clinical efficacy was the improvement in the subjective symptoms of patients, such as numbness, coldness and pain, and a decreased MDNS [14].

### 2.2. DPN Diagnostic Criteria

This study referred to the DPN diagnostic criteria [1] as follows: history of diabetes; numbness, pain, chills, burning, or other abnormal feelings as the main symptoms; an electromyogram indicating that the nerve conduction speed had slowed down or that the latency period was prolonged; and a MDNS indicating nerve damage.

### 2.3. Diagnostic Criteria for Qi-Yin Deficiency Syndrome

According to the “Guiding Principles for Clinical Research of New Chinese Medicine in the Treatment of Diabetes” [14] and the Diabetes Branch of the Chinese Medicine Association “Guidelines for TCM Clinical Diagnosis and Treatment of DPN” [15], the criteria for qi-yin deficiency syndrome are as follows: numbness; pain (cold or burning); electric shock; tingling; pain at night; pins and needles feeling; tiredness; dry skin; a sense of decreased pain; a dark, purple, or spotted tongue; and a fine or astringent pulse.

### 2.4. Inclusion Criteria

The inclusion criteria were as follows: patients who met the DPN diagnostic criteria with qi-yin deficiency syndrome and patients whose medical records, prescription medication data, and other information were complete.

### 2.5. Exclusion Criteria

The following patients were excluded: patients with peripheral neuropathy caused by other diseases; patients who were unwilling to cooperate (could not cooperate with dietary control or did not use medicines as prescribed); patients with severe acute or chronic complications of diabetes or other serious primary diseases; patients who did not take CHM regularly; or patients for whom clinical efficacy could not be evaluated.

### 2.6. Treatment

Patients followed the diabetes diet, exercised regularly, and took CHM in the morning and evening based on the conventional treatment for DPN.

### 2.7. Establishment of the Database and Regulating Data

Information on age, sex, body mass index (BMI), MDNS, and biochemical indexes of DPN patients with qi-yin deficiency syndrome was entered into an Excel database. The CHM database was established using the “Diabetes Clinical Medical Case Diagnosis and Treatment Information Collection System” developed by The First Affiliated Hospital of Anhui University of Chinese Medicine. This database was base on Xuezhong Zhou's platform [16]. This platform promotes the development of TCM from individualized empirical knowledge to evidence-based medicine.

Medical records were entered by trained physicians and then checked by the quality control staff of the research team to ensure the accuracy of the information. The names of CHM prescriptions were standardized according to the 2015 edition of the “Pharmacopoeia of the People's Republic of China” [17].

### 2.8. Statistical Analyses

Statistical analyses were performed using SPSS 23.0 statistical software (SPSS Inc., Chicago, IL, USA). Measurement data with a normal distribution are expressed as the mean ± standard deviation (SD), and the squared deviation method was used for cluster analysis. The CHM rules were analysed with the a priori algorithm, entropy clustering of complex systems, and Bayesian network analysis. The prediction accuracy of the machine learning classification methods including association rules, cluster analysis, and complex networks using clinical data were 84.1%, 75.0%, and 84.5%, respectively [16, 18]. *P* < 0.05 was considered to indicate statistical significance.

## 3. Results

### 3.1. Comparison of Baseline Characteristics

**Figure 1:**
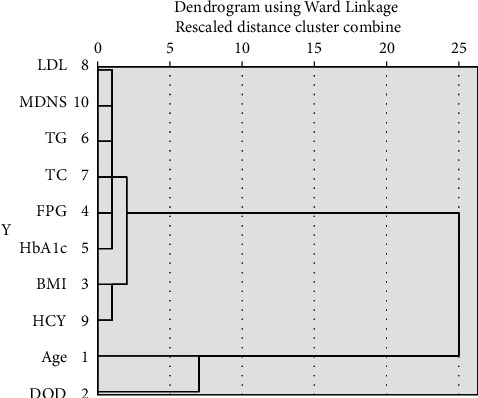
Cluster analysis from baseline characteristics.

Of the 216 patients, 152 patients were male, and 64 patients were female. The baseline characteristics of patients are shown in Table 1. The data were divided into two groups by duration of diabetes (DOD) of 10 years or more, and the characteristics of two groups are shown in Table 2. Cluster analysis was performed on the baseline characteristics. When the abscissa was set to 10, data for low-density lipoprotein cholesterol (LDL-C), MDNS, triglycerides (TG), total cholesterol (TC), fasting plasma glucose (FPG), glycated haemoglobin (HbA1c), age, DOD, BMI, and homocysteine (HCY) were obtained. The results are shown in Figure 1.

### 3.2. Typical Syndrome Characteristics

**Figure 2:**
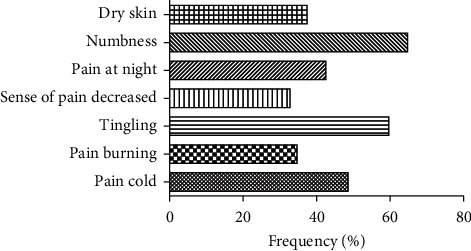
Typical syndrome characteristics.

There were 162 patients who had typical syndrome characteristics of DPN and 54 patients who had no symptoms. The frequencies of the typical syndrome characteristics are shown in Figure 2. Among the characteristics, numbness occurred most often.

### 3.3. CHM Frequency

A total of 182 types of CHM were used, and the top 20 types are listed in Table 3. *Astragalus membranaceus* was the most frequently used, with a frequency of 67.12% in 216 prescriptions. Among the CHM, more than 45% were *Ligusticum wallichii*, *Poria cocos*, and Radix Rehmanniae. The highest dosage of *Polygonatum kingianum* Coll. et Hemsl was 28 g.

### 3.4. CHM Characteristics

There were 64 warm herbals, 59 cold, 34 neutral, 10 cool, and 5 hot.

The following flavours were included: 94 were sweet, 80 were bitter, 59 were spicy, 17 were sour, 9 were light, 8 were salty, and 6 were astringent.

There were 80 herbals for liver meridians, 72 for lung meridians, 67 for spleen meridians, 62 for kidney meridians, 40 for heart meridians, 18 for large bowel meridians, 14 for bladder meridians, 10 for small intestine meridians, 5 for pericardium meridians, and 3 for triple energizer meridians.

### 3.5. Core Prescriptions

**Figure 3:**
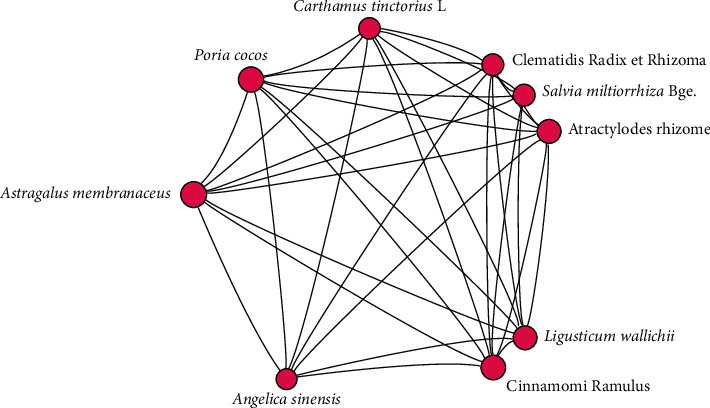
Core prescriptions.

Cluster analysis was performed on all CHM. The 9 most frequently CHM were *Astragalus membranaceus*, *Ligusticum wallichii*, *Poria cocos*, atractylodes rhizome, *Salvia miltiorrhiza* Bge., *Angelica sinensis*, Cinnamomi Ramulus, *Carthamus tinctorius* L., and Clematidis Radix et rhizome. The results are shown in Figure 3.

### 3.6. CHM Correlation

Correlation analysis was performed on all CHM. The support was >80%, and the confidence was >90%. There were 5 groups of CHM pairs, and there were 3 groups of CHM triads with support ≥ 75% and confidence ≥ 95%. The results are shown in Table 4.

### 3.7. CHM Cluster

**Figure 4:**
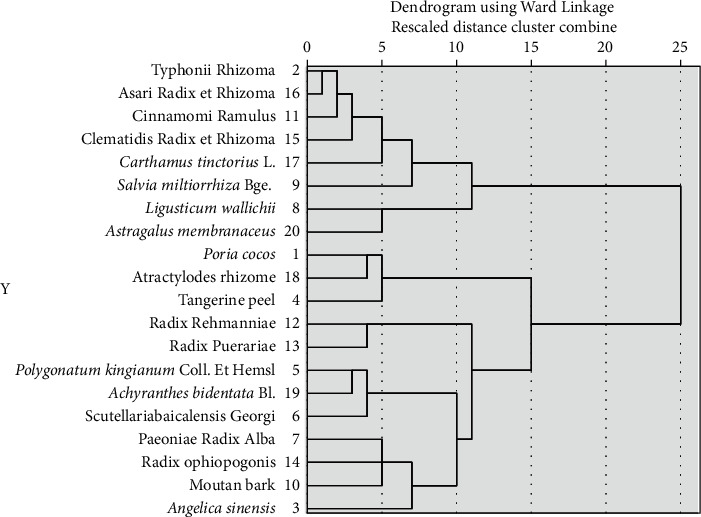
Cluster analysis from CHM.

Cluster analysis was performed on 20 types of CHM in terms of frequency. When the abscissa was set to 5, data on Typhonii Rhizoma-Asari Radix et Rhizoma-Cinnamomi Ramulus-Clematidis Radix et Rhizoma-*Carthamus tinctorius* L., *Ligusticum wallichii*-*Astragalus membranaceus*, *Poria cocos*-atractylodes rhizome-Tangerine peel, Radix Rehmanniae-Radix Puerariae, *Polygonatum kingianum* Coll. et Hemsl-*Achyranthes bidentata* Bl.-*Scutellaria baicalensis* Georgi, and Paeoniae Radix Alba-Radix Ophiopogonis-Moutan bark were obtained. The results are shown in Figure 4.

### 3.8. CHM and Correlation Indicators

The analysis showed that CHM can lead to recognized evidence of improved microindicators by the application of association rules to mine correlations between CHM herbs and indicators. The results are shown in Table 5.

## 4. Discussion

HbA1c is an important indicator of glycaemic control among patients with diabetes. For most adult patients with diabetes, a reasonable target for HbA1c is <7.0%. Studies have shown that when HbA1c levels are >8.0%, the prevalence of DPN increases significantly [19]. HbA1c levels in this study were approximately 9.91%, indicating that the glycaemic control of the patients was not good. Therefore, a high level of HbA1c is one of the main reasons that patients develop DPN with qi-yin deficiency syndrome. According to the comparison based on a DOD that had reached 10 years, patients with a long duration of disease had decreased HbA1c levels, likely due to the strict control of glycaemic as a result of fear of diabetic complications.

In this study, cluster analysis was performed on the baseline characteristics of patients to obtain 3 groups, including patients with average age and DOD. Some studies have shown that patients with a DOD of more than 10 years often have DPN [20]. However, the average DOD in this study was 84 months; thus, DPN may have been diagnosed earlier when the patient exhibited clinical manifestations than in patients with a DOD of 10 years. Other studies may have included asymptomatic patients. The average HCY levels in this study were higher than the normal range. Bruce and Young [21] and El Boghdady and Badr [22] found that a high HCY is an important risk factor for DPN. High HCY levels can affect the functions of insulin sensitivity, oxidative stress, nitric oxide, and other pathways of blood vessel and blood vessels and nerves damage [23, 24]. Therefore, HCY may be an important reference value for the early diagnosis of DPN. However, some scholars believe that HCY is not related to DPN [25]. We found that symptoms due to DPN had a frequency of 75%, which was higher than the findings reported in previous studies, which ranged from 16% to 65% [26, 27]. This difference may be because the patients were hospitalized, indicating that their conditions were more serious.

This study included 216 patients with DPN with qi-yin deficiency syndrome. The results showed the top 5 CHM that were prescribed frequently: *Astragalus membranaceus*, *Ligusticum wallichii*, *Poria cocos*, Radix Rehmanniae, and atractylodes rhizome. Among these herbals, 3 were warm, 1 was cold, and 1 was neutral. Based on these 5 CHM, or 64 warm CHM out of 216 prescriptions, it is believed that the treatment of DPN should be focused on warm CHM. *Astragalus membranaceus* is sweet and warm. Moreover, it nourishes qi and promotes blood circulation. Furthermore, it is widely used in the treatment of DPN, and it is a monarch CHM. Astragaloside IV and polysaccharides in *Astragalus membranaceus* decrease glycaemic and regulate blood lipids [28]. Moreover, the antioxidant effect of *Astragalus membranaceus* improves the symptoms caused by nerve damage [29]. In addition, the dose of *Astragalus membranaceus* is relatively large, which is consistent with the findings of Feng et al. [30]. Radix Rehmanniae is sweet and cold. It nourishes yin, promotes blood circulation, and moisturizes the skin. Its administration may offer an opportunity to correct epithelial progenitor cell EPC deficits in diabetic patients with damaged blood vessels and nerves [31]. When used clinically, the dose should not be too high to avoid producing a cold or upset stomach. Radix Rehmanniae is a minister and assistant CHM.

These 9 CHM comprise the core of the 216 prescriptions. *Astragalus membranaceus* is the most core herbal. Cinnamomi Ramulus warms the meridians to stimulate blood to reach the limbs, and it is a minister and assistant CHM. Cinnamaldehyde is the main component of Cinnamomi Ramulus and decreases glycaemic, improves microcirculation, and mediates antiplatelet aggregation and analgesia effects [32]. *Astragalus membranaceus* and Cinnamomi Ramulus have significant effects on pain in the limbs. This theory may be derived from the application described in the Han Dynasty [33].

The 8 pairs of CHM were identified through correlation analysis. Codonopsis Radix replenishes qi and promotes blood circulation, so that patients do not feel pain or numbness. Radix Ophiopogonis acts by cultivating yin. Analysis of the pharmacological effects of Codonopsis Radix and Radix Ophiopogonis indicates that they decrease glycaemic and are antioxidative [34, 35]. These effects may relieve clinical DPN symptoms. *Astragalus membranaceus* and atractylodes rhizome are essential medicines for Yupingfeng powder. *Astragalus membranaceus* has the effect of two-way regulation of excessive sweat and reduced sweat. These two herbals may also have good clinical effect on antioxidation through polysaccharides in Yupingfeng power [36]. This may regulate abnormal skin sweating in DPN.

Six sets of CHM were identified from the cluster analysis. *Poria cocos* and atractylodes rhizome are a typical companion CHM pair that improve microcirculatory effects, desensitize channel blocking action to nicotinic acetylcholine receptors, and have antineuronal differentiation actions [37, 38]. These two CHM also prevent emotional depression or hyperactivity caused by DPN symptoms. If patients are in obvious pain, Asari Radix et Rhizoma and Typhonii Rhizoma can be added to relieve pain because these herbals exert sedative, analgesic, and anti-inflammatory effects [39].

There are a series of associations between CHM and indicators. *Astragalus membranaceus* and Cinnamomi Ramulus or *Ligusticum wallichii* and Moutan bark are highly related to a decreased MDNS. Feldman et al. [40] reported that the sensitivity and specificity of the clinical portion of the MDNS are 80% and 95%, respectively. The AUC for MDNS scores was 0.906 [41], the MDNS is a good screening tool for diabetic neuropathy, *Astragalus membranaceus*, Cinnamomi Ramulus, and *Ligusticum wallichii* are included in the Huangqi Guizhi Wuwu decoction. Pang et al. [42] and Liang et al. [43] performed a systematic review and meta-analysis reporting that this decoction improves DPN symptoms and ameliorates nerve conduction velocities. However, their studies did not explicitly mention the MDNS. Jin et al. studied the improvement in the MDNS caused by TCM [44]. Radix Puerariae or *Poria cocos* is highly related to decreased FPG and HbA1c levels when combined with Radix Rehmanniae and Radix Ophiopogonis or *Astragalus membranaceus*. Lin et al. [35] reported that alcoholic and acetic acid fermentation improve the hypoglycaemic activity of Radix Ophiopogonis. Radix Puerariae has been reported to have antidiabetic effects [45], and these crude herbals reduce the magnitude of increases in glycaemic [46]. Pharmacological studies have indicated that *Astragalus membranaceus* possesses antioxidant and antidiabetic effects [47]. *Poria cocos*, as a prebiotic, can potentially prevent or cure metabolic diseases by regulating lipids and glycaemic [48]. If combined with other herbs, *Poria cocos* makes the hypoglycaemic effect more obvious, which can further regulate HbA1c. Naqvi et al. [49] reported that LDL-C is a related risk factor for DPN. Based on the control of cardiovascular disease risk factors in the context of diabetes and its complications, reduction of LDL-C is recommended as an important goal. In this study, *Astragalus membranaceus* was associated with *Poria cocos* when LDL-C was decreased.

This study preliminarily assessed the basic characteristics of DPN patients with qi-yin deficiency syndrome and summarized the characteristics of TCM treatment through data mining technology. In the future, the sample size will be increased for in-depth verification of the findings to improve clinical evidence and to better guide clinical practice. The mechanism of action of these CHM prescriptions can also be further explored [50].

## Figures and Tables

**Table 1:** Baseline characteristics.

Variable	Value (mean ± SD)
Age (year)	62.74 ± 11.25
DOD (month)	84.19 ± 62.20
BMI (kg/m^2^)	25.16 ± 4.78
FPG (mmol·L^−1^)	8.54 ± 4.01
HbA1c (%)	9.91 ± 2.86
TG (mmol/L)	1.94 ± 1.76
TC (mmol/L)	4.59 ± 1.03
LDL-C (mmol/L)	2.59 ± 1.03
HCY (cm)	18.98 ± 22.40
MDNS (fraction)	25.84 ± 1.12

**Table 2:** Comparison of baseline characteristics in the two groups by DOD.

Variable	≥10 years	<10 years	Value	*P* value
Male/female	42/21	110/43	0.585	0.512
Age (year)	64.73 ± 11.77	61.92 ± 10.97	1.674	0.096
BMI (kg/m^2^)	24.70 ± 4.36	25.35 ± 4.95	−0.916	0.361
FPG (mmol·L^−1^)	8.00 ± 3.91	8.75 ± 4.04	−1.253	0.212
HbA1c (%)	9.26 ± 2.63	10.17 ± 2.91	−2.140	0.033*∗*
TG (mmol/L)	1.75 ± 1.42	2.02 ± 1.87	−1.015	0.311
TC (mmol/L)	4.44 ± 0.94	4.64 ± 1.06	−1.342	0.181
LDL-C (mmol/L)	2.46 ± 1.07	2.63 ± 1.00	−1.094	0.275
HCY (cm)	17.79 ± 18.99	19.47 ± 23.71	−0.051	0.617
MDNS (fraction)	25.73 ± 5.25	25.89 ± 6.06	−0.061	0.951

**Table 3:** CHM frequencies and dose.

Drug name	Frequency (%)	Dose (g)	Drug name	Frequency (%)	Dose (g)
*Astragalus membranaceus*	145 (67.12)	24	Tangerine peel	74 (34.26)	12
*Ligusticum wallichii*	109 (50.46)	15	Radix Puerariae	64 (29.63)	16
*Poria cocos*	106 (49.07)	17	Asari Radix et Rhizoma	61 (28.24)	6
Radix Rehmanniae	99 (45.83)	16	Radix Ophiopogonis	54 (25.00)	23
Atractylodes rhizome	97 (44.90)	19	Paeoniae Radix Alba	53 (24.54)	25
*Salvia miltiorrhiza* Bge.	89 (41.20)	10	Moutan bark	52 (24.07)	22
*Angelica sinensis*	88 (40.74)	10	*Scutellaria baicalensis* Georgi	50 (23.15)	13
Cinnamomi Ramulus	86 (39.81)	22	Typhonii Rhizoma	49 (22.68)	6
*Carthamus tinctorius* L.	78 (36.11)	14	*Achyranthes bidentata* Bl.	39 (18.05)	26
Clematidis Radix et Rhizoma	77 (35.64)	27	*Polygonatum kingianum* Coll. et Hemsl	39 (18.05)	28

**Table 4:** CHM correlation.

CHM pair		Support (%)	Confidence (%)
After item	Before item		
Codonopsis pilosula	Radix Ophiopogonis	81.82	96.38
*Scutellaria baicalensis* Georgi	Radix Rehmanniae	80.63	95.32
*Poria cocos*	Atractylodes rhizome	82.84	93.88
*Ligusticum wallichii*	*Angelica sinensis*	81.87	92.64
Tangerine peel	Paeoniae Radix Alba	84.16	90.51
*Rehmannia*	*Astragalus membranaceus* and atractylodes rhizome	84.25	98.65
*Cornus officinalis* Sieb. et Zucc.	*Astragalus membranaceus* and atractylodes rhizome	76.86	97.27
Moutan bark	*Astragalus membranaceus* and Paeoniae Radix Alba	80.83	96.04

**Table 5:** CHM and correlation indicators.

Drug	Indicators	Support (%)	Confidence (%)
*Astragalus membranaceus* and Cinnamomi Ramulus	MDNS ↓	56.74	77.35
*Ligusticum wallichii* and Moutan bark	MDNS ↓	50.25	74.31
Radix Rehmanniae, Radix Ophiopogonis, and Radix Puerariae	FPG and HbA1c ↓	32.43	55.21
*Astragalus membranaceus* and *Poria cocos*	FPG and HbA1c ↓	23.15	49.36
*Salvia miltiorrhiza* Bge. and Pinelliae Rhizoma	LDL-C ↓	20.35	62.73

## Data Availability

The data used to support the findings of this study are included within the article.
